# Diagnosis and treatment of uncommon ileal endometriosis: a case report and literature review

**DOI:** 10.52054/FVVO.13.4.046

**Published:** 2021-12-30

**Authors:** M Mabrouk, D Raimondo, M Cofano, L Cocchi, R Paradisi, R Seracchioli

**Affiliations:** Division of Gynaecology and Human Reproduction Physiopathology, Department of Medical and Surgical Sciences (DIMEC), IRCCS Azienda Ospedaliero, Univeristaria di Bologna. S. Orsola Hospital, University of Bologna, Via Massarenti 13, Bologna, 40138 Italy; Cambridge Endometriosis & Endoscopic Surgery Unit (CEESU) and Cambridge University Hospitals NHS Foundation Trust, Addenbrooke’s Hospital, Cambridge, United Kingdom

**Keywords:** ileal endometriosis, endometriosis, small bowel endometriosis, laparoscopic surgery, laparoscopy

## Abstract

Endometriosis is defined as the presence of endometrial tissue outside the uterine cavity. It is a common finding in premenopausal women and commonly affects the gastrointestinal tract, especially the rectosigmoid tract. Small bowel involvement is rare and usually asymptomatic making diagnosis difficult. Here we report an uncommon case of exophytic ileal endometriosis surgically treated. Detailed pre-operative counselling on the risk of ileal surgery should always be considered in all cases with endometriosis requiring surgery. We also present a review of the literature regarding the clinical presentation, diagnosis, and treatment of this challenging condition.

## Introduction

Endometriosis is a chronic, inflammatory, oestrogen-dependent disease defined as the presence of endometrial glands and stroma outside the uterine cavity and it affects up to 15% of women during reproductive age (Olive and Pritts, 2001). The most frequent implants are the ovaries, uterosacral ligaments, cul-de-sac, vagina, urinary tract, and bowel. (Chapron et al., 2006; Chapron et al., 2003) In women with endometriosis, bowel involvement is relatively common (3-12%) and the most frequent localisations occur in the rectum and rectosigmoid junction (50-90%), the small bowel (2-16%), appendix (3-18%) and caecum (2-5%). The ileum is affected in 4% of women with bowel endometriosis, (Chapron et al., 2006) frequently associated with rectosigmoid lesions.

Women with isolated ileal endometriosis are usually asymptomatic or have non-specific symptoms: abdominal pain, bloating and cramps, altered bowel habits (constipation and/or diarrhea), dyspareunia and haematochezia (Rodriguez-Lopez et al., 2018). There is no high-precision imaging test for ileal endometriosis (Tong et al., 2013). Diagnosis of ileal endometriosis is usually made incidentally during surgery for other endometriosis sites or following direct complications of ileal involvement: bowel obstruction, ileocecal intussusception or perforation (Rodriguez-Lopez et al., 2018). At laparoscopy, typical ileal lesions are nodular in shape with transmural and endophytic growth and are often located on the ileocecal junction (Ruffo et al., 2011).

Here we present, for the first time, an asymptomatic exophytic nodule of the distal ileum as an uncommon case of ileal endometriosis.

## Case history

A 41-year-old multiparous woman presented to our centre with a history of severe dysmenorrhea, dyspareunia and catamenial dysuria within the last six months resistant to progestin therapy. She also reported intermittent perimenstrual intestinal bloating and constipation. She had recently undergone a diagnostic laparoscopy in another hospital and was diagnosed with bladder endometriosis. Gynaecological examination revealed moderate pain on anterior and posterior vaginal fornix palpation. Transvaginal sonography (TVS) and magnetic resonance imaging (MRI) showed the presence of adenomyosis and a deep endometriotic nodule of the left uterosacral ligament (USL) (17 mm mean diameter) and bladder dome (25 mm mean diameter).

Surgery was planned for the excision of the endometriotic nodule by partial cystectomy and removal of the affected left USL. Because of her intestinal symptoms, the woman was informed and gave informed consent about the risk of bowel surgery, including small bowel and appendix procedures.

**Figure 1:**
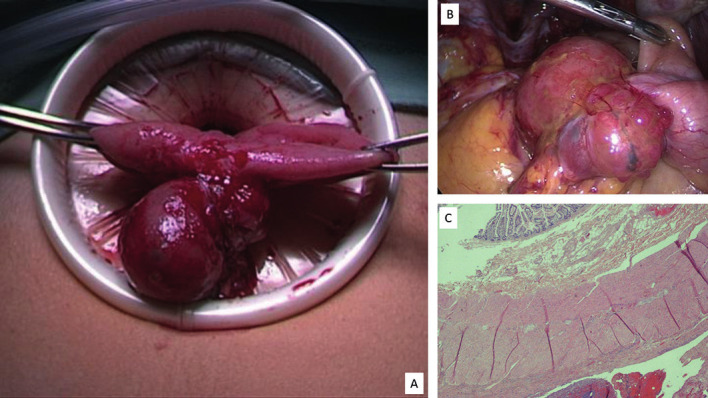
A: Mini-laparotomy with extrusion of the distal ileum affected by an endometriotic nodule. B: Diagnostic laparoscopy showing a 4 cm round exophytic mass of 4 cm with a smooth, translucent-blue surface coated by small nodules on the distal ileum. C: Ileal endometriosis, at histological examination, 10x magnification. Note an area of florid endometriosis in the ileal tract serosa. The mucosa with villi is opposite and is not involved by endometriosis and phlogosis. On the serosa there is an intense and active phlogosis with hemorrhagic spread.

At diagnostic laparoscopy we systematically perform, before inspecting the pelvis, an assessment of the abdomen, including appendix, ileocaecal junction, and distal ileum. During the inspection, a 4 cm round, exophytic mass with a translucent-blue and smooth surface covered with small nodules on the distal ileum was found (Figure 1). Ileal surgery was performed by an experienced multidisciplinary team with general surgeon and urologist. After removal of the pelvic lesions, mobilisation of the caecum and distal ileum a transverse suprapubic mini- laparotomy were performed to externalise the ileum.

The affected bowel tract was isolated and resected, and side-to-side anastomosis was performed with the GIA75 device. The postoperative course was regular. Pathological examination confirmed a 4-cm exophytic ileal endometriotic implant without atypical features and endometriosis of the appendix. At 6-month follow-up, pain and bowel symptoms markedly improved. The women provided informed consent to report data and images of the case.

## Review of literature and Discussion

We performed a narrative review on endometriosis diagnosis and management. To determine eligible articles, the following electronic databases were screened from inception to January 2021: PubMed, Scopus, and Embase. To retrieve articles related with the theme of interest, the following terms were used to search the electronic databases: “endometriosis” AND “ile*” OR “small bowel”. Reports included in this review consisted of case reports and clinical studies describing management of ileal endometriosis. Two study investigators (M.C. and L.C.) independently conducted the primary literature research using the main search terms.

A total of 12 studies (7 case report, 5 clinical studies) were selected and included in this systematic review (Table I).

**Table I:** Ileal Endometriosis: literature review

Author, year	Cases (n)	Median age (range)	Endometriosis previous diagnosis	Main Symptoms	Indication for surgery	Size mm (range)	Performed imaging	Diagnostic method	Associated endometriotic lesion	Ileal surgical procedure	Surgical approach	Complications	Median follow up time (range)	After surgery
De Ceglie et al., 2008	1	44	No	Diffuse abdominal pain, diarrhoea alternating constipation	Acute small bowel obstruction	50 mm	US,colonoscopy, CT	CT (irregular mass involving the ileum)	No	Right hemicolectomy and distal ileum resection	LPT	NS	NS	NS
Ruffo et al., 2011	31	34 (25 - 40)	22 (71%)	CPP, Constipation and dyschezia	Severe pelvic pain (100%)	NS	US, BDCE, CT	Intraoperative finding (100%)	Colorectal endometriosis (29 cases-94%)	Ileocecal resection (100%)	LPS (100%)	1:reoperation for bleeding; 4 (13%) blood cells transfusion; 1 ureteral fistula	27 months (12-56) Available for 18 pz	12: normal defecation 4: mild constipation presented before surgery 2: altered bowel habits 16:regression/improvements of pain
Karaman et al., 2012	1	27	No	Longstanding abdominal pain and diarrhoea	Acute small bowel obstruction	NS	Colonoscopy, small bowel endoscopy, CT	Histological examination	Left ovarian cyst	Distal ileum resection with end-ileostomy, left cystectomy	LPT	No	NS	NS
Tong et al., 2013	1	41	No	Recurrent abdominal pain, vomiting, diarrhoea	Acute small bowel obstruction	50x50 mm	Colonoscopy, CT	CT	No	Ileocecal resection with end-to-end anastomosis	LPT	No	12 months	No recurrence of symptoms
Rousset et al., 2014	6	NS	NS	Abdominal cramping or pain, bloating, diarrhea, constipation, nausea or vomiting, symptoms of bowel obstruction	Pelvic DIE	NS	3,0-T MRI Enterography	3,0-T MRI Enterography (100%)	Rectum or rectosigmoid junction (5 – 83%)	Ileocecal resection (100%)	NS	NS	6 months	Resolution and no recurrence of symptoms
Fedele et al., 2014	8	29-43	6 (75%)	8: severe CPP 6: constipation and dyschezia	Pelvic pain (7 - 88%); acute small bowel obstruction (1 – 12%)	NS	US, 2: BDCE, 6: colonoscopy	1: BDCE 1: colonoscopy	Rectosigmoid lesions (7- 88%), rectovaginal lesion (1 – 12%)	Ileocecal resection (100%)	LPT (100%)	1: blood transfusion 1: permanent right femoral nerve lesion	106 +/- 10 months	8: significant improvement of pelvic pain 3: mild-moderate pain 2: constipation 2: alternating constipation al loose stool 1: bloating
Gimonet et al., 2016	4	34 (18-61)	4 (36.3%)	Dysmenorrhea, dyspareunia, abdominal pain, dyschezia, constipation, rectorrhagia	NS	NS	MRI	NS	NS	Ileocecal resection	NS	NS	NS	NS
Bacalbasa et al., 2017	1	37	Yes	Acute diffuse abdominal pain, vomiting	Acute small bowel obstruction	NS	US, CT	Intraoperative finding	Bilateral ovarian cysts and rectosigmoid lesion	Right ileocolectomy with ileocolic anastomosis, rectosigmoid resection and bilateral cystectomy	NS	No	NS	NS
Chan et al., 2017	1	46	No	2 days history of vomiting, abdominal distension, absolute constipation	Acute small bowel obstruction	NS	CT	Intraoperative finding	NS	Distal ileum resection with end-to-end anastomosis	LPS	NS	NS	NS
López Carrasco et al., 2017	7	35 (30-41)	4 (57.1 %)	4: CPP 3: catamenial pseudo-obstruction 1: acute bowel obstruction 1: intestinalpseudo-occlusion	NS	NS	NS	3: MRI and BDCE 1: MRI 3: intraoperative finding	3: Rectosigmoid 4: USL	Ileal resection and end-to-end anastomosis(100%)	5 cases: LPS 2 cases: LPT	1:dehiscence of rectal anastomosis and postoperative hemorrhage	NS	Improvement of painful symptoms (100%)
Imasogie et al., 2018	1	42	NO	Constipation, abdominal pain, abdominal swelling, anorexia, easy satiety	Small Bowel pseudo-obstruction	60x30x35 mm	NS	Intraoperative finding	No	Right hemicolectomy with ileotransverseanastomosis	LPT	NS	NS	NS
Ungerleider et al., 2019	1	52	NO	Purulent cutaneous drainage from right lower abdomen	Spontaneous enterocutaneous fistula in the right lower abdomen	70x70x50 mm	CT	CT	No	Ileal resection	LPT	NS	NS	NS

Notes: US: Ultrasound; LPS: Laparoscopy; LPT: Laparotomy; CT: computerized tomographic; BDCE: double contrast barium enema; MRI: Magnetic Resonance Imaging; CPP: Chronic Pelvic Pain; DIE: deep infiltrating endometriosis; USL: Uterosacral Ligament, NS: not specified.

We found a total of 63 cases of ileal endometriosis. Age ranged from 18 to 61 years.

Out of 56 women with available data about surgical history for endometriosis, 37 (66%) had a previous diagnosis.

Symptoms were variously reported among studies. Particularly, abdominal pain and chronic pelvic pain were the most described ones, occurring in 46/53 (87%) women. Bowel symptoms were present in 23/46 (50%) women. Seven women suffered of small bowel obstruction. Clinical presentation was detailed only in five cases: 3 presented longstanding story of diarrhoea and abdominal pain, while 2 acute onset with abdominal pain and vomiting.

Primary indications for surgery, when reported, were pelvic pain in 38 (73%) women, acute small bowel obstruction in 7 (13%), diagnosis of pelvic deep infiltrating endometriosis (DIE) in 6 (12%), spontaneous enterocutaneous fistula in one (2%).

Concerning the preoperative imaging, several imaging modalities were used. Specifically, of the 37 women who underwent computerised tomography (CT), an ileal lesion was suspected in only 3 (8%) of them. Rousset et al. (2014) diagnosed all 6 cases of ileocaecal endometriotic lesions using 3.0-Tesla (T) MRI enterography; conversely, in Gimonet et al. (2016) no ileal lesion were found using MRI in 6 patients. A preoperative diagnosis of ileal endometriosis was suspected in one (3%) of 33 women who had double contrast barium enema (BDCE) and in one of the 9 women (11%) who underwent colonoscopy.

Diagnosis of ileal endometriosis was made during surgery in 43 women (43/63 – 68,3%), using 3.0-T MRI enterography in 6 cases (6/63 – 9,5%), using MRI in 4 cases (4/63 – 6,3%), using CT in 3 cases (3/63 – 4,7%), in 1 case with BDCE (1,6%), in 1 case with colonoscopy, and in 1 case only at histological examination.

The presence of other lesions of DIE during surgery was found in 50 of 58 women (86%).

The surgical approach varies between the studies and were not always specified. Laparoscopic approach was performed in 37 women of 52 (71%), while 15 women underwent laparotomy (29%).

Only six studies investigated surgical complications. Out of 49 women with available data, 5 cases (10%) required blood cell transfusion, one (2%) reoperation for bleeding, one ureteral fistula, one permanent right femoral nerve lesion and one rectal fistula.

Among women with available follow-up, 95% (38/40) reported improvement of pain symptoms. In Ruffo et al. (2011) 2 women (2/18, 11%) reported altered bowel habits; in Fedele et al. (2014) two patients (2/8, 25%) complained constipation, two (2/8 - 25%) alternating constipation and loose stool, and one bloating (1/8 - 13%).

Due to the rarity of cases, the literature regarding small bowel endometriosis is limited to a few case reports. The first case of ileal endometriosis was published in 1956 by G. Melody, (Melody, 1956) after which several isolated cases were reported (Table 1).

Bowel endometriosis lesions can result from the implantation of ectopic endometrial cells on the intestinal serosa that progressively invades the bowel wall. Endometriosis infiltrating the muscularis propria of the bowel can lead to localised fibrosis in the bowel wall, stenosis and bowel obstruction (Anaf et al., 2004). Small bowel endometriosis may be suspected in nulliparous fertile women with signs of bowel obstruction, especially during menstruation (Orbuch et al., 2007). However, women with isolated ileal endometriosis may be asymptomatic or present with non-specific/overlapped symptoms.

While the diagnosis of rectosigmoid endometriosis can often be obtained through rectovaginal examination, TVS, transrectal sonography and MRI, the diagnosis of ileal endometriosis was more challenging (Fedele et al., 2014). MRI had the highest sensitivity (77%-93%) for the diagnosis of bowel endometriosis, especially for lesions with endophytic growth, however, this sensitivity declined considering only the small bowel (Bazot et al., 2004).

In all cases, particularly in those with non- specific/overlapped symptoms, careful diagnostic laparoscopy mapping all potential locations of endometriosis in the abdomino-pelvic cavity is essential to prevent repetitive surgeries and relative complications. On abdominal inspection, usually ileal lesions are nodular in shape with transmural and endophytic growth. If small bowel endometriosis is present, laparoscopic ileal/ileocecal resection should be considered part of complete eradication of macroscopic disease, also because this endometriosis is mainly constituted of ﬁbrosis and sclerosis, which do not respond to hormonal treatment (Ruffo et al., 2011).

This is the first reported case of exophytic endometriotic ileal nodule.

## Conclusions

Since most cases of ileal endometriosis are overlooked during preoperative work-up, detailed pre-operative counselling on the risk of ileal/ ileocecal resection is mandatory, in symptomatic cases with endometriosis requiring surgery. Furthermore, having a high level of clinical suspicion for ileal endometriosis is also important to optimise the diagnostic accuracy of imaging technique and diagnostic laparoscopy.
